# Supplementary material for: Epidermal growth factor receptor (EGFR) and vascular endothelial growth factor A (VEGF-A) expressions in Ethiopian female breast cancer and their association with histopathologic features
Source: PLoS One. 2024 Oct 15;19(10):e0308411. doi: 10.1371/journal.pone.0308411 (PMC11478813; doi:10.1371/journal.pone.0308411)
Supplement: Attachment — Submitted filename: Manuscript - EGFR & VEGF- PLOS ONE - Edited Final.doc [file pone.0308411.s001.doc]

**EGFR and VEGF-A expressions in Ethiopian female breast cancer and their**

**Association with histopathologic features “don’t use abbreviation in the title, if possible, try to indicate your main finding in the title”**

Sisay Addisu^1*^, Abebe Bekele^2#a^, Mathewos Assefa^3^, Tuffa Gemechu^4^, Mark J. Hoenerhoff^5^, and Sofia D. Merajver^6^

*^1^Department of Biochemistry, School of Medicine, College of Health Sciences, Addis Ababa University, Addis Ababa, Ethiopia*

*^2^Department of Surgery, School of Medicine, College of Health Sciences, Addis Ababa University, Addis Ababa, Ethiopia*

*^3^Department of Internal Medicine, School of Medicine, College of Health Sciences, Addis Ababa University, Addis Ababa, Ethiopia*

*^4^Department of Pathology, School of Medicine, College of Health Sciences, Addis Ababa University, Addis Ababa, Ethiopia*

*^5^In Vivo Animal Core, Unit for Laboratory Animal Medicine, University of Michigan Medical School, Ann Arbor, Michigan, United States of America*

*^6^Division of Hematology and Oncology, Department of Internal Medicine, University of Michigan Rogel Cancer Center, Ann Arbor, Michigan, United States of America.*

^#a^ Current Address: School of Medicine, University of Global Health Equity, Kigali, Rwanda

*Corresponding Author:

E-mail: sisay.addisu@aau.edu.et

***ABSTRACT***

**Background:** Epidermal growth factor receptor (EGFR) and vascular endothelial growth factor receptor (VEGF) play important role in breast tumor growth, invasion, metastasis, patient survival and drug resistance. The aim of this study was to evaluate the protein expression status of EGFR and VEGF-A, as well as their association with hormone receptor status and histopathological characteristics in the invasive type of female breast cancer among Ethiopians.

**Method:** The primary breast tumor tissues were obtained from 85 Ethiopian invasive breast cancer cases that underwent modified radical mastectomy (MRM) from June 2014 to June 2015. Their FFPE blocks were analyzed for EGFR and VEGF protein expressions using immunohistochemical techniques. The expressions were also correlated with histopathologic features.

**Result:** EGFR over-expression was observed in 22% of the tumor samples. VEGF-A expression was negative in 13.41%, low in 63.41%, moderate in 20.73%, and high in 2.44%. EGFR expression, but not VEGF-A, showed a significant inverse correlation with both ER (*P* = 0.01) and PR (*P* = 0.04). EGFR and VEGF expressions did not show significant association with tumor size, grade, lymph node status or age at diagnosis.

**Conclusion:** EGFR expression was most likely associated with ER and PR negative tumors. Assessments of multiple molecular markers aid to understand the biological behavior of the disease in Ethiopian population. It might also help to predict which group of patients might get more benefit from the selected treatment strategies and which are not.

**Key words:** EGFR, VEGF, immunohistochemistry

**INTRODUCTION**

Breast cancer is a complex disease that exhibits variations in clinical, morphological and biological characteristics [1]. Currently, five treatment options are used to address breast cancer: surgery, radiotherapy, chemotherapy, hormonal therapy and immunotherapy. The selection of treatments is based on the grade, stage and molecular subtype of the cancer [2]. Surgery, chemotherapy, and radiotherapy are commonly used for all types of breast cancer, while endocrine therapies are employed for hormone receptor-positive cases and anti-HER2 therapy for HER2+ cases, targeting specific molecular subtypes [2, 3]. Despite recent advancements in targeted breast cancer treatment, challenges persist in the form of therapeutic resistance, disease recurrence, and metastasis, influenced by other signaling molecules and pathways [4, 5]. Among other factors, the expression of epidermal growth factor receptor (VEGF) and vascular endothelial growth factor receptor (EGFR) has been shown to play a role in therapeutic resistance against breast cancer treatment.

EGFR plays a significant role in carcinogenesis due to its oncogenic activity through various mechanisms [6, 7]. Previous studies have reported an association between EGFR expression and a poor response to tamoxifen treatment in breast cancer. The signaling cross-talk between ER and growth factor receptor pathways leads to resistance against hormonal therapy [8, 9]. EGFR expression is also associated with poor tumor differentiation, ER negativity, a higher mitotic index, and decreased survival [10]. Increased expression of EGFR is also associated with resistance to anti-HER2-targeted therapy in HER2+ breast cancer cases [11]. Anti-EGFR therapies may benefit individuals with EGFR overexpressing breast cancer and could serve as a predictive marker for EGFR targeted treatments. However, despite the numerous preclinical and clinical trials investigating anti-EGFR inhibitors in breast cancer, only two of them, Lapatinib and Neratinib, have been approved by the Federal Drug Administrations (FDA). Lapatinib, an anti HER1/HER2 targeted drug, is approved for hormone positive and HER2-positive breast cancer. On the other hand, Neratinib, an anti HER1, HER2, and HER4 tyrosine-protein kinase inhibitor, is approved for HER2 positive breast cancer under specific conditions [12, 13, 14].

VEGF family members have been recognized to play fundamental roles by activating their receptors and have also been shown to be overexpressed in breast cancer [15, 16]. Among them, VEGF-A has been identified as the most potent angiogenic factor, inducing proliferation, and migration in endothelial cells. Additionally, it promotes tumor cell proliferation, survival and migration [15]. Higher expression of VEGF-A in breast cancer cases has been significantly correlated with poor prognosis and shorter overall survival in some studies [17, 18, 19]. Additionally, VEGF expression has been associated with resistance to hormonal therapy in hormone-receptor positive breast cancer cases [20]. VEGF targeted therapies such as Bevacizumab and angiogenesis-related tyrosine kinase inhibitors were approved for the treatment of various malignancies [3]. However, the antitumor response to Bevacizumab in breast cancer patients was not as expected, leading to a revision of the FDA decision due to inconclusive results regarding progression-free and overall survival benefits. There is currently no approved VEGF-targeted therapy for the treatment of breast cancer [16, 20,21].

Breast cancer is the most common type of cancer in Ethiopia [22]. However, there is limited information available about its clinical and pathobiological characteristics. To the best of our knowledge, no study has reported on the status of VEGF and EGFR expression in Ethiopian female breast cancer. This study is the first of its kind to explore the expression of VEGF and EGFR, as well as their association with hormone receptors and clinico-pathological features in Ethiopian females with invasive ductal carcinoma of the breast.

**3. MATERIALS AND METHODS**

**Study participants and tumor samples:** The present study involved female patients with clinically and pathologically confirmed invasive breast carcinoma. These patients underwent MRM, which included axillary lymph node dissection, between June 2014 and June 2015. The resected tissues were sent to two referral hospitals for morphological analysis and FFPE preparation. Some FFPE blocks were excluded from the study due to reasons such as being undetectable or having non-invasive tumors. Ultimately, 85 female patients with invasive breast cancer who met the inclusion criteria and had sufficient histopathologic data were included in the study.

Due to inadequate clinical information such as clinical stage, menopausal status and family history, patient data were obtained from the standard histopathology requesting and reporting forms. The information gathered included age at diagnosis, tumor size, histologic type, tumor grade and lymph node status. Immunohistochemical biomarkers, including ER, PR, EGFR, and VEGF-A, were examined at the University of Michigan. Pathologic grading was based on the Nottingham combined histological grade [23]. Pathologic classifications of axillary lymph node involvement and tumor size were determined according to the American Joint Committee on Cancer (AJCC) definition [24].

***Immunohistochemical Analysis:*** Immunohistochemical analysis was conducted using deparaffinized sections of FFPE tumor tissues. The sections were deparaffinized, rehydrated, and subjected to epitope retrieval using Dako PT Link. Primary antibodies for ER (clone SP1, Rabbit monoclonal, Cell Marque, Rocklin, CA), PR (clone Y85, Rabbit monoclonal, Cell Marque, Rocklin, CA), EGFR (clone 31G7, Mouse monoclonal, Invitrogen, Camarillo, CA) and VEGF (A-20 SC-152, Rabbit polyclonal, Santa Cruz, CA) were applied using automated immunostainer (Dako Autostainer Link, Carpintaria, CA, USA). After applying specific monoclonal antibodies (for ER, PR and EGFR) and polyclonal antibody (VEGF), FLEX+ Mouse/Rabbit EnVision Systems (Agilent, Dako) were used for detection. The IHC slides were then scanned using the Aperio slide scanning system (Aperio Technologies, Inc., Vista, CA).

***Immunohistochemical evaluation:*** The evaluation of the IHC stained slides of ER and PR was conducted using Allred’s scoring system [25]. The percentage of positive tumor cells was graded on a scale of 0-5, and the intensity of labeling was graded on a scale of 0-3. The total score was calculated by adding the proportion and intensity scores, resulting in a range of 0-8 (0-2 labeled as negative and 3-8 labeled as positive).

The score for EGFR protein expression was reported based on previous publications [26, 27]. EGFR staining was considered positive when at least 10% of tumor cells exhibited strong membranous staining. Staining in normal adjacent tissues and stromal labeling was not considered as positive staining. For the evaluation of VEGF expression level, a semi-quantitative scoring system was used, which combined scores for the proportion of tumor cells stained positive and the intensity of the staining. This scoring system was previously described by Dhakal et al. [28] (see table 1).

**Table 1. Scoring system for VEGF-A protein expression**

| **% Positive Cells** | **Proportion Score** | **Intensity** | **Intensity Score** |
| --- | --- | --- | --- |
| 0 | 0 | No staining of tumor cells | 0 |
| ≤10% of positive cells | 1 | Weak staining | 1 |
| 10-50% of positive tumor cells | 2 | Moderate staining | 2 |
| >50% of positive tumor cells | 3 | Strong staining | 3 |

***Ethical Considerations:*** Ethical approval was obtained from the Department Ethical Review Committee (DERC), the Institutional Review Board (IRB) of the College of Health Sciences at Addis Ababa University, and the Federal Health Research Ethics Review Committee of the Ministry of Science and Technology in Ethiopia.

***Statistical Analysis:*** We conducted statistical analysis of our data using SPSS 24.0 software (SPSS Inc., Chicago, IL, USA). Descriptive statistics were used to summarize the frequency distributions. The Pearson’s chi-square test was employed to determine associations between categorical variables. Fischer’s exact test was used as necessary. A two-tailed P value was of < 0.05 was considered statistically significant for the results.

**RESULTS**

***Patient and tumor characteristics:***

The age distribution of the patients ranged from 22 to 75 years (mean ± standard deviation (SD), 42.14 ± 11.96). The frequency percentages of the age groups for the 85 study participants and their tumor histopathologic characteristics are shown in Table 2.

Based on histologic subtype, 89% (74/83) of primary breast cancer samples were identified as IDC type. More than 60% (49/80) of our cancer samples belong to T3 and T4. Similarly, more than 70% (57/77) of the breast cancer tumors showed axillary lymph node metastasis. Among the histologically assessed tumor grades, 51% (36/71) belonged to poorly differentiated tumors (grade III) and 38% (27/71) were moderately differentiated (grade II) tumors. The lymph node status of 14 cases in our study samples was not assessed.

Regarding the status of hormonal receptors, the majority of the study samples showed positive expressions for ER (73.8%) and PR (60.2%). Membranous EGFR staining was observed in varying percentages of tumor cells, ranging from 0 to 99%. Specifically, 17 cases (20.5%) had an expression of less than 10%, 6 cases (7.2%) had an expression between 10% and 25%, 1 case (1.2%) had an expression between 50% and 90%, 6 cases had an expression greater than 90%, and 48 cases (57.8%) showed no expression. Tumors with strong in more than 10% of the tumor cells were considered EGFR positive, which was observed in only 18 cases (21.7%).

VEGF-A expression was calculated by multiplying the proportion and intensity scores with a range of 0 to 9. Scores from 0 to 3 were categorized as no ⁄low expression, 4 to 6 as moderate expression, and greater than 6 as high expression. In this study VEGF-A expression was observed as high in 2 (2.4%), moderate in 17 cases (20.5%) at moderate concentration, low in 53 cases (63.9%) at a low concentration, and negative in 11 cases (13.3%).

***Association of EGFR and VEGF-A with Clinicopathological Features***

The correlations between the overexpression of EGFR and clinicopathological features are shown in Table 3. EGFR overexpression showed a significant association with ER (P=0.01) and PR (P=0.04). However, EGFR overexpression was not significantly associated with age, tumor size, histological grade, and axillary lymph node involvement.

In this study, tumors with moderate or strong VEGF expressions were categorized as positive, while the rest were considered negative for comparison with other categorical variables. VEGF-A expression was not significantly associated with any of the studied parameters (Table 4).

**Table 2. Clinicopathological features**

| Characteristics | Count | Percentage frequency (%) |
| --- | --- | --- |
| **Age** |  |  |
| <50 | 60 | 70.6 |
| ≥50 | 25 | 29.4 |
| **Histological subtype** |  |  |
| Invasive Ductal Carcinoma | 74 | 89.2 |
| Invasive Lobular Carcinoma | 3 | 3.6 |
| Mixed ductal and Lobular Carcinoma | 3 | 3.6 |
| Mucinious carcinoma | 2 | 2.4 |
| Papillary carcinoma | 1 | 1.2 |
| Missing | 2 |  |
| **Tumor Size** |  |  |
| T1 | 9 | 11.3 |
| T2 | 22 | 27.5 |
| T3 | 26 | 32.5 |
| T4 | 23 | 28.8 |
| Missing | 5 |  |
| **Histological Grade** |  |  |
| G1 | 8 | 11.3 |
| G2 | 27 | 38.0 |
| G3 | 36 | 50.7 |
| Missing | 14 |  |
| **Lymph Node Status** |  |  |
| N0 | 20 | 26.0 |
| N1 | 30 | 39.0 |
| N2 | 23 | 29.9 |
| N3 | 4 | 5.2 |
| Missing | 8 |  |
| **ER status** |  |  |
| Positive | 62 | 73.8 |
| Negative | 22 | 26.2 |
| Missing | 1 |  |
| **PR status** |  |  |
| Positive | 50 | 60.2 |
| Negative | 33 | 39.8 |
| Missing | 2 |  |
| **EGFR status** |  |  |
| Positive | 18 | 21.7 |
| Negative | 65 | 78.3 |
| Missing | 2 |  |
| **VEGF status** |  |  |
| High | 2 | 2.4 |
| Moderate | 17 | 20.5 |
| Low | 53 | 63.9 |
| Negative | 11 | 13.3 |
| Missing |  |  |

**Table 3. Association of EGFR overexpression with clinicopathological features.**

| **Characteristics** | **EGFR** | |  |
| --- | --- | --- | --- |
|  | **Positive, N (%)** | **Negative, N (%)** | p-Value |
| **Age**  <50  ≥50 | 14 (24.1)  4 (16.0) | 44 (75.9)  21 (84.0) | 0.41 |
| **ER**  Positive  Negative | 9 (14.5)  9 (42.9) | 53 (85.5)  12 (57.1) | **0.01** |
| **PR**  **Positive**  **Negative** | 7 (14.0)  11 (33.3) | 43 (86.0)  22 (66.7) | **0.04** |
| **Tumor size** pT1-pT2  pT3-pT4 | 6 (20.0)  12 (25.0) | 24 (80.0)  36 (75.0) | 0.61 |
| **Histological grade**  G1/G2  G3 | 5 (14.7)  10 (28.6) | 29 (85.3)  25 (71.4) | 0.16 |
| **Lymph node status**  pN0-pN1  pN2-pN3 | 12 (24.5)  5 (18.5) | 37 (75.5)  22 (81.5) | 0.55 |

**Table 4. Association of EGFR and VEGF protein expressions with histopathological features.**

| **Characteristics** | **VEGF** |  |  |
| --- | --- | --- | --- |
|  | **Positive, N (%)** | **Negative, N (%)** | p-Value |
| **Age**  <50  ≥50 | 11 (19.0)  8 (32.0) | 47 (81.0)  17 (68.0) | 0.19 |
| **ER**  Positive  Negative | 16 (25.8)  3 (14.3) | 46 (74.2)  18 (85.7) | 0.28 |
| **PR**  Positive  Negative | 11 (22.0)  8 (25.0) | 39 (78.0)  24 (75.0) | 0.75 |
| **Tumor size**  pT1-pT2  pT3-pT4 | 7 (22.6)  12 (25.5) | 24 (77.4)  35 (74.5) | 0.77 |
| **Histological grade**  G1/G2  G3 | 8 (22.9)  7 (20.6) | 27 (77.1)  27 (79.4) | 0.82 |
| **Lymph node status**  pN0-pN1  pN2-pN3 | 10 (20.4)  7 (26.9) | 39 (79.6)  19 (73.1) | 0.52 |

**DISCUSSION**

To our knowledge, this study is the first of its kind to assess the EGFR and VEGF status among female invasive breast cancer patients in the Ethiopian population. Generally, studies have reported an overexpression of EGFR ranging from 16% to 36% in breast cancer cases [29]. In our study, 21.7% of the tumor samples tested positive for EGFR. This finding is similar to the European population (20.6%) [30], but lower than the Tunisian population (28.6%) [31] and Black Americans (25.2%), while higher than White Americans (17.5%) [32]. It is important to note that different studies have reported varying results, even within the same country, due to the lack of standardized scoring systems [33, 34, 35, 36, 37]. Therefore, the implementation of a standardized protocol may be necessary for future Ethiopian breast cancer studies to accurately assess EGFR status.

In the current study, a significant inverse association was observed between hormonal receptor status and EGFR expression, which is consistent with previous studies [32, 33, 38, 39]. This may be due to the down-regulation of hormone receptor expression by EGFR signaling [40, 41, 42]. EGFR has been indicated to be a potential drug target for triple-negative breast cancer cases [43]. On the other hand, ER is used as the primary therapeutic target for hormone receptor positive patients. However, EGFR overexpression has also been associated with resistance to endocrine treatment in this group [44]. Targeting EGFR in addition to endocrine treatment could potentially delay drug resistance and improve the survival rate of hormone receptor positive breast cancer cases [45].

Although EGFR positivity was more frequent in younger age cases (40% vs. 24%), the current study failed to show a significant association, which is in agreement with many studies [31, 38, 46, 47, 48]. Previous studies have reported a significant association between EGFR expression and younger age or premenopausal breast cancer cases [32, 38]. The variation in results compared to previous studies may be attributed to the small sample size or differences in the measurement technique for EGFR expression. Our study also did not show a significant association with tumor size and lymph node status, which is consistent with previous studies [33, 38, 47, 48-50]. However, a significant association of EGFR positivity with tumor size and lymph node status was reported in other studies [32, 51]. The variation in results compared to previous studies may be due to small sample size, differences in tissue processing, the type of assay used for EGFR measurement, and the cut-off points for EGFR positivity. The current study also showed a higher frequency of EGFR positivity among grade III tumors compared to grade I/II tumors, although this difference was not statistically significant. On the other hand, other studies have reported a significant association of EGFR over-expression with high-grade tumors in invasive types of breast carcinoma [31, 32, 48, 52]. The lack of significant association between EGFR expression and tumor grade in the current study may be attributed to differences in the selection of study groups, pre-analytical procedures, the type of primary antibodies used, the score used for EGFR interpretation, and the small sample size.

The majority of the tumors in the current study showed positive expression of VEGF-A (84.4%), which is similar to previous studies [53, 54]. The positivity rate of VEGF-A in this study was higher compared to other reports [55, 56]. However, Cimpean et al (2008) reported an increased frequency of VEGF positivity [57]. Dhakal et al (2012) also reported a higher frequency of moderate VEGF-A expression in Norwegians compared to the current study [28]. The variations in positivity rate may be attributed to differences in sample size, pre-analytical procedures, type of primary antibodies used for detection, scoring criteria for VEGF positivity or genetic polymorphism [58-62].

In the current study, no significant association was observed between expressions of VEGF and hormone receptors, which is consistent with several previous studies [56, 61, 63-65]. However, other studies have reported inverse correlations [66, 67]. The lack of association in our study may be attributed to the small sample size, differences in types of cases, scoring criteria for VEGF positivity, and variation in VEGF detection techniques. Co-expression of hormone receptors and VEGF-A has been shown to be associated with shorter recurrence-free and overall survival even after adjuvant endocrine treatment [67, 68].

Our study did not find a significant association between VEGF expression and age, which is consistent with previous studies [55, 56, 61, 63, 67, 69]. However, other studies have reported significant associations with conflicting results [19, 66]. Therefore, further investigations are needed to understand the association between VEGF expression and the age of patients.

The present study did not show a significant association between VEGF-A expression and histopathological features (tumor size, histologic grade and axillary lymph node status), which is consistent with previous studies [50, 61, 70]. However, other studies have reported a significant association with lymph node status [56, 67]. Furthermore, conflicting results have been reported regarding the association with tumor size [56, 67, 71-73] and grade [63, 67, 74, 75]. The variation in results between this study and previous studies may be attributed to differences in sample size, tissue fixation and processing, types of VEGF-A clones, scoring system for positivity and dichotomization of categorical variables. Additionally, the lack of association between VEGF expression and histopathological features in our study may also be due to the minimal role of VEGF-A in autocrine signaling as a result of reduced expression of VEGFR2 (the major VEGF-A receptor) by the tumor cells [76], or partly due to increased secretion of other angiogenic factors such as fibroblast growth factor, which contributes to tumor growth [77]. Although the expression of VEGFRs by breast tumor cells is controversial, studies have found low expression of VEGFR2 by breast tumor cells [78-80].

The current study has some limitations. The first limitation is the small sample size and the expression of high VEGF-A levels in only a small proportion of the tumor samples. These limitations may restrict the ability of the current study to draw definitive conclusions. Secondly, because the status of HER-2 expressions is unknown, the frequency of TNBC and its association with VEGF-A and EGFR could not be determined. Thirdly, this study did not include prospective or retrospective observations. Therefore, the prognostic values of the study parameters were not assessed.

**CONCLUSION**

In the current study, there was a significant association between EGFR expression and hormone receptor status. This suggests that EGFR could be a reliable predictor for adjuvant therapy in cases of hormone receptor negative breast cancer. Although it is difficult to draw definitive conclusion due to other unstudied parameters, EGFR might be one of the potential therapeutic targets for this group. Moreover, EGFR overexpressing ER negative patients may benefit from currently approved anti-HER1/HER2 therapies based on their condition if they fail to respond to other, more effective treatments. Despite its limitations, this study lays the groundwork for future breast cancer studies in the Ethiopian population. Therefore, we recommend conducting further research with a sufficient sample size, comprehensive clinical information and well-designed methodologies.

**Acknowledgments**

We would like to express our gratitude to the University of Michigan African Studies Center, the University of Michigan African Presidential Scholars (UMAPS) program, the University of Michigan Comprehensive Cancer Center, and Addis Ababa University for their technical and material support. We also extend our thanks to Professor Senait Fisseha from the University of Michigan for her generous assistance in obtaining a funding and ensuring the practicality of this work.

**Declarations**

**Ethical approval and informed consent**

The study protocol was reviewed and approved by the Biochemistry Department Ethics and Research Review Committee (DERC), the Institutional Review Board (IRB) of the College of Health Sciences at Addis Ababa University, and the Federal Health Research Ethics Review Committee of the Ministry of Science and Technology in Ethiopia. A written informed consent statement was obtained from each study participant.

**Competing interests**

The authors declare that they have no competing interests regarding the publication of this paper.

**Authors' contributions**

All authors equally contribute to the study design, technical and material support, analysis and supervision.

**Funding**

This research was supported by the Center for International Reproductive Health Training (CIRHT) program at the University of Michigan.

**Availability of data and materials**

The datasets generated and analyzed in this study are available upon request from the corresponding author.

**REFERENCES**

1. Rakha EA, Tse GM, Quinn CM. An update on the pathological classification of breast cancer. Histopathology. 2023;82(1):5-16.
2. Burguin A, Diorio C, Durocher F. Breast Cancer Treatments: Updates and New Challenges. J Pers Med. 2021;11(8):808.
3. Wang Y, Minden A. Current Molecular Combination Therapies Used for the Treatment of Breast Cancer. Int J Mol Sci. 2022;23(19):11046.
4. Arpino G, Wiechmann L, Osborne CK, Schiff R: Crosstalk between the estrogen receptor and the her tyrosine kinase receptor family: Molecular mechanism and clinical implications for endocrine therapy resistance. Endocr Rev 2008;29:217-233.
5. Giuliano M, Schifp R, Osborne CK, Trivedi MV: Biological mechanisms and clinical implications of endocrine resistance in breast cancer. Breast 2011;20:S42-49.
6. Thomas R, Weihua Z. Rethink of EGFR in Cancer With Its Kinase Independent Function on Board. Front Oncol. 2019;9:800.
7. Uribe ML, Marrocco I, Yarden Y. EGFR in Cancer: Signaling Mechanisms, Drugs, and Acquired Resistance. Cancers (Basel). 2021;13(11):2748.
8. García-Becerra R, Santos N, Díaz L, Camacho J. Mechanisms of resistance to endocrine therapy in breast cancer: focus on signaling pathways, miRNAs and genetically based resistance. Int J Mol Sci. 2012;14(1):108-45.
9. Siatis KE, Giannopoulou E, Manou D, Sarantis P, Karamouzis MV, Raftopoulou S, Fasseas K, Alzahrani FM, Kalofonos HP, Theocharis AD. Resistance to hormone therapy in breast cancer cells promotes autophagy and EGFR signaling pathway. Am J Physiol Cell Physiol. 2023;325(3):C708-C720.
10. Masuda H, Zhang D, Bartholomeusz C, Doihara H, Hortobagyi GN, Ueno NT. Role of epidermal growth factor receptor in breast cancer. Breast Cancer Res Treat. 2012;136(2):331-45.
11. Luque-Cabal M, García-Teijido P, Fernández-Pérez Y, Sánchez-Lorenzo L, Palacio-Vázquez I. Mechanisms Behind the Resistance to Trastuzumab in HER2-Amplified Breast Cancer and Strategies to Overcome It. Clin Med Insights Oncol. 2016;10(Suppl 1):21-30.
12. Iancu G, Serban D, Badiu CD, Tanasescu C, Tudosie MS, Tudor C, Costea DO, Zgura A, Iancu R, Vasile D. Tyrosine kinase inhibitors in breast cancer (Review). Exp Ther Med. 2022;23(2):114.Top of Form
13. Chaurasia M, Singh R, Sur S, Flora SJS. A review of FDA approved drugs and their formulations for the treatment of breast cancer. Front Pharmacol. 2023;14:1184472.
14. Zubair T, Bandyopadhyay D. Small Molecule EGFR Inhibitors as Anti-Cancer Agents: Discovery, Mechanisms of Action, and Opportunities. Int J Mol Sci. 2023;24(3):2651.
15. Al Kawas H, Saaid I, Jank P, Westhoff CC, Denkert C, Pross T, Weiler KBS, Karsten MM. How VEGF-A and its splice variants affect breast cancer development - clinical implications. Cell Oncol (Dordr). 2022;45(2):227-239.
16. Ayoub NM, Jaradat SK, Al-Shami KM, Alkhalifa AE. Targeting Angiogenesis in Breast Cancer: Current Evidence and Future Perspectives of Novel Anti-Angiogenic Approaches. Front Pharmacol. 2022;13:838133.
17. Arias-Pulido H, Chaher N, Gong Y, Qualls C, Vargas J, Royce M. Tumor stromal vascular endothelial growth factor A is predictive of poor outcome in inflammatory breast cancer. BMC Cancer. 2012 ;12:298.
18. Hodorowicz-Zaniewska D, Kibil W, Małek A, Szpor J, Kulig J, Sztefko K. Evaluation of serum concentrations of vascular endothelial growth factor (VEGF) in breast cancer patients. Pol J Pathol. 2012;63(4):255-60.
19. Sa-Nguanraksa D, O-Charoenrat P. The role of vascular endothelial growth factor a polymorphisms in breast cancer. Int J Mol Sci. 2012;13(11):14845-64.
20. Brogowska KK, Zajkowska M, Mroczko B. Vascular Endothelial Growth Factor Ligands and Receptors in Breast Cancer. J Clin Med. 2023;12(6):2412.
21. Karsten MM, Beck MH, Rademacher A, Knabl J, Blohmer JU, Jückstock J, Radosa JC, Jank P, Rack B, Janni W. VEGF-A165b levels are reduced in breast cancer patients at primary diagnosis but increase after completion of cancer treatment. Sci Rep. 2020;10(1):3635.
22. Sung et al., 2021 - Sung, H.; Ferlay, J.; Siegel, R.L.; Laversanne, M.; Soerjomataram, I.; Jemal, A.; Bray, F. Global cancer statistics 2020: GLOBOCAN estimates of incidence and mortality worldwide for 36 cancers in 185 countries. CA Cancer J. Clin. 2021; 71: 209–249.
23. Elston CW, Ellis IO. Pathological prognostic factors in breast cancer. I. The value of histological grade in breast cancer: experience from a large study with long-term follow-up. Histopathology. 1991; 19: 403–410.
24. [Giuliano AE,](https://www.ncbi.nlm.nih.gov/pubmed/?term=Giuliano%2520AE%255BAuthor%255D&cauthor=true&cauthor_uid=28294295) [Connolly JL,](https://www.ncbi.nlm.nih.gov/pubmed/?term=Connolly%2520JL%255BAuthor%255D&cauthor=true&cauthor_uid=28294295) [Edge SB,](https://www.ncbi.nlm.nih.gov/pubmed/?term=Edge%2520SB%255BAuthor%255D&cauthor=true&cauthor_uid=28294295) [Mittendorf EA,](https://www.ncbi.nlm.nih.gov/pubmed/?term=Mittendorf%2520EA%255BAuthor%255D&cauthor=true&cauthor_uid=28294295) [Rugo HS,](https://www.ncbi.nlm.nih.gov/pubmed/?term=Rugo%2520HS%255BAuthor%255D&cauthor=true&cauthor_uid=28294295) [Solin LJ,](https://www.ncbi.nlm.nih.gov/pubmed/?term=Solin%2520LJ%255BAuthor%255D&cauthor=true&cauthor_uid=28294295) [Weaver DL,](https://www.ncbi.nlm.nih.gov/pubmed/?term=Weaver%2520DL%255BAuthor%255D&cauthor=true&cauthor_uid=28294295) [Winchester DJ,](https://www.ncbi.nlm.nih.gov/pubmed/?term=Winchester%2520DJ%255BAuthor%255D&cauthor=true&cauthor_uid=28294295) [Hortobagyi GN.](https://www.ncbi.nlm.nih.gov/pubmed/?term=Hortobagyi%2520GN%255BAuthor%255D&cauthor=true&cauthor_uid=28294295) Breast cancer-major changes in the American Joint Committee on Cancer eighth edition cancer staging manual. [CA Cancer J Clin.](https://www.ncbi.nlm.nih.gov/pubmed/28294295) 2017; 67(4): 290-303.
25. [Fitzgibbons PL,](https://www.ncbi.nlm.nih.gov/pubmed/?term=Fitzgibbons%2520PL%255BAuthor%255D&cauthor=true&cauthor_uid=24236805) [Dillon DA,](https://www.ncbi.nlm.nih.gov/pubmed/?term=Dillon%2520DA%255BAuthor%255D&cauthor=true&cauthor_uid=24236805) [Alsabeh R,](https://www.ncbi.nlm.nih.gov/pubmed/?term=Alsabeh%2520R%255BAuthor%255D&cauthor=true&cauthor_uid=24236805) [Berman MA,](https://www.ncbi.nlm.nih.gov/pubmed/?term=Berman%2520MA%255BAuthor%255D&cauthor=true&cauthor_uid=24236805) [Hayes DF,](https://www.ncbi.nlm.nih.gov/pubmed/?term=Hayes%2520DF%255BAuthor%255D&cauthor=true&cauthor_uid=24236805) [Hicks DG,](https://www.ncbi.nlm.nih.gov/pubmed/?term=Hicks%2520DG%255BAuthor%255D&cauthor=true&cauthor_uid=24236805) [Hughes KS,](https://www.ncbi.nlm.nih.gov/pubmed/?term=Hughes%2520KS%255BAuthor%255D&cauthor=true&cauthor_uid=24236805) [Nofech-Mozes S.](https://www.ncbi.nlm.nih.gov/pubmed/?term=Nofech-Mozes%2520S%255BAuthor%255D&cauthor=true&cauthor_uid=24236805) Template for reporting results of biomarker testing of specimens from patients with carcinoma of the breast. [Arch Pathol Lab Med.](https://www.ncbi.nlm.nih.gov/pubmed/?term=Template+for+reporting+results+of+biomarker+testing+of+specimens+from+patients+with+carcinoma+of+the+breast.) 2014; 138(5): 595-601.
26. Kim MJ, Ro JY, Ahn SH, Kim HH, Kim SB, Gong G. Clincopathologic significance of the basal-like subtype of breast cancer: a comparison with hormone receptor and Her2/neu-overexpressing phenotypes. Hum Pathol. 2006; 37(9): 1217-1226.
27. Mokhtari M, Ardestani MM, Movahedipour M. An immunohistochemical study of EGFR expression in colorectal cancer and its correlation with lymph nodes status and tumor grade. J Res Med Sci. 2012;17 (8):741-744.
28. Dhakal HP, Naume B, Synnestvedt M, Borgen E, Kaaresen R, Schlichting E, Wiedswang G, Bassarova A, Holm R, Giercksky KE, Nesland JM. Expression of vascular endothelial growth factor and vascular endothelial growth factor receptors 1 and 2 in invasive breast carcinoma: prognostic significance and relationship with markers for aggressiveness. Histopathology. 2012; 61: 350-64.
29. Zeng T, Gao H, Yu P, He H, Ouyang X. Up-Regulation of Kin17 is essential for proliferation of breast cancer. PLOS ONE. 2011; 6(9): e25343.
30. Park K, Han S, Shin E, Kim HJ, Kim JY. EGFR gene and protein expression in breast cancers. Eur J Surg Oncol. 2007; 33: 956–960.
31. [Kallel I,](https://www.ncbi.nlm.nih.gov/pubmed/?term=Kallel I%5BAuthor%5D&cauthor=true&cauthor_uid=22394363) [Khabir A,](https://www.ncbi.nlm.nih.gov/pubmed/?term=Khabir A%5BAuthor%5D&cauthor=true&cauthor_uid=22394363) [Boujelbene N,](https://www.ncbi.nlm.nih.gov/pubmed/?term=Boujelbene N%5BAuthor%5D&cauthor=true&cauthor_uid=22394363) [Abdennadher R,](https://www.ncbi.nlm.nih.gov/pubmed/?term=Abdennadher R%5BAuthor%5D&cauthor=true&cauthor_uid=22394363) [Daoud J,](https://www.ncbi.nlm.nih.gov/pubmed/?term=Daoud J%5BAuthor%5D&cauthor=true&cauthor_uid=22394363) [Frikha M,](https://www.ncbi.nlm.nih.gov/pubmed/?term=Frikha M%5BAuthor%5D&cauthor=true&cauthor_uid=22394363) [Aifa S,](https://www.ncbi.nlm.nih.gov/pubmed/?term=Aifa S%5BAuthor%5D&cauthor=true&cauthor_uid=22394363) [Sallemi-Boudawara T,](https://www.ncbi.nlm.nih.gov/pubmed/?term=Sallemi-Boudawara T%5BAuthor%5D&cauthor=true&cauthor_uid=22394363) [Rebaï A.](https://www.ncbi.nlm.nih.gov/pubmed/?term=Rebaï A%5BAuthor%5D&cauthor=true&cauthor_uid=22394363) EGFR overexpression relates to triple negative profile and poor prognosis in breast cancer patients in Tunisia. [J Recept Signal Transduct Res.](https://www.ncbi.nlm.nih.gov/pubmed/?term=EGFR+overexpression+relates+to+triple+negative+profile+and+poor+prognosis+in+breast+cancer+patients+in+Tunisia) 2012;32(3):142-9.
32. Rimawi MF, Shetty PB, Weiss HL, Schiff R, Osborne CK, Chamness GC, Elledge RM. Epidermal growth factor receptor expression in breast cancer association with biologic phenotype and clinical outcomes. Cancer. 2010: 116 (5): 1234–1242.
33. Magkou C, Nakopoulou L, Zoubouli C, Karali K, Theohari I, Bakarakos P, Giannopoulou I. Expression of the epidermal growth factor receptor (EGFR) and the phosphorylated EGFR in invasive breast carcinomas. Breast Cancer Res. 2008; 10(3): R49.
34. Koletsa T, Kotoula V, Karayannopoulou G, Nenopoulou E, Karkavelas G, Papadimitriou CS, Kostopoulos I. EGFR expression and activation are common in HER2 positive and triple-negative breast tumours. Histol Histopathol. 2010; 25: 1171-1179.
35. Shao MM, Zhang F, Meng G, Wang XX, Xu H, Yu XW, Chen LY, Tse GM. Epidermal growth factor receptor gene amplification and protein overexpression in basal-like carcinoma of the breast. Histopathology. 2011; 59: 26473.
36. Altaf FJ, Mokhtar GA, Emam E, Bokhary RY, Mahfouz NB, Al Amoudi S, Al-Gaithy ZK. Metaplastic carcinoma of the breast: an immunohistochemical study. Diagn Pathol. 2014; 9: 139.
37. Nakajima H, Ishikawa Y, Furuya M, Sano T, Ohno Y, Horiguchi J, Oyama T. Protein expression, gene amplification, and mutational analysis of EGFR in triple-negative breast cancer. Breast Cancer. 2014; 21(1): 66-74.
38. Zheng Z, Shao N, Weng H, Li W, Zhang J, Zhang L, Yang L, Ye S. Correlation between epidermal growth factor receptor and tumor stem cell markers CD44/CD24 and their relationship with prognosis in breast invasive ductal carcinoma. Med Oncol. 2015; 32: 275.
39. Kinnel B, Singh SK, Oprea-Ilies G, Singh R. Targeted Therapy and Mechanisms of Drug Resistance in Breast Cancer. Cancers (Basel). 2023 Feb 19;15(4):1320. doi: 10.3390/cancers15041320. PMID: 36831661; PMCID: PMC9954028.
40. Skasko E, Paszko Z, Mazur S. A new look at the prognostic value of the estrogen, progesterone and epidermal growth factor receptors in breast cancer tissue. Neoplasma. 2005; 52(1): 10–17.
41. [Dowsett M,](https://www.ncbi.nlm.nih.gov/pubmed/?term=Dowsett%2520M%255BAuthor%255D&cauthor=true&cauthor_uid=16497822) [Houghton J,](https://www.ncbi.nlm.nih.gov/pubmed/?term=Houghton%2520J%255BAuthor%255D&cauthor=true&cauthor_uid=16497822) [Iden C,](https://www.ncbi.nlm.nih.gov/pubmed/?term=Iden%2520C%255BAuthor%255D&cauthor=true&cauthor_uid=16497822) [Salter J,](https://www.ncbi.nlm.nih.gov/pubmed/?term=Salter%2520J%255BAuthor%255D&cauthor=true&cauthor_uid=16497822) [Farndon J,](https://www.ncbi.nlm.nih.gov/pubmed/?term=Farndon%2520J%255BAuthor%255D&cauthor=true&cauthor_uid=16497822) [A'Hern R,](https://www.ncbi.nlm.nih.gov/pubmed/?term=A'Hern%2520R%255BAuthor%255D&cauthor=true&cauthor_uid=16497822) [Sainsbury R,](https://www.ncbi.nlm.nih.gov/pubmed/?term=Sainsbury%2520R%255BAuthor%255D&cauthor=true&cauthor_uid=16497822) [Baum M.](https://www.ncbi.nlm.nih.gov/pubmed/?term=Baum%2520M%255BAuthor%255D&cauthor=true&cauthor_uid=16497822) Benefit from adjuvant tamoxifen therapy in primary breast cancer patients according oestrogen receptor, progesterone receptor, EGF receptor and HER2 status. [Ann Oncol. 2](https://www.ncbi.nlm.nih.gov/pubmed/16497822)006; 17(5): 818-26.
42. Chen XS, Wang KS, Guo W, Li LY, Yu P, Sun XY, Wang HY, Guan YD, Tao YG, Ding BN, Yin MZ, Ren XC, Zhang Y, Chen CS, Ye YC, Yang JM, Cheng Y. UCH-L1-mediated Down-regulation of Estrogen Receptor α Contributes to Insensitivity to Endocrine Therapy for Breast Cancer. Theranostics. 2020;10(4):1833-1848.
43. Ma J, Dong C, Cao YZ, Ma BL. Dual Target of EGFR and mTOR Suppresses Triple-Negative Breast Cancer Cell Growth by Regulating the Phosphorylation of mTOR Downstream Proteins. Breast Cancer (Dove Med Press). 2023;15:11-24.
44. Jeong Y, Bae SY, You D, Jung SP, Choi HJ, Kim I, Lee SK, Yu J, Kim SW, Lee JE, Kim S, Nam SJ. EGFR is a Therapeutic Target in Hormone Receptor-Positive Breast Cancer. Cell Physiol Biochem. 2019;53(5):805-819.
45. Hanker AB, Sudhan DR, Arteaga CL. Overcoming Endocrine Resistance in Breast Cancer. Cancer Cell. 2020;37(4):496-513.
46. Ansquer Y, Mandelbrot L, Lehy T, Salomon L, Dhainaut C, Madelenat P, Feldmann G, Walker F. Expression of BRCA1, HER-1 (EGFR) and HER-2 in sporadic breast cancer and relationships to other clinicopathological prognostic features. Anticancer Res. 2005;25(6C):4535-41.
47. Ji Z, Yang L, Ruan Q. Correlation of epidermal growth factor receptor (EGFR), androgen receptor (AR) and 14-3-3 sigma expression in breast cancer. Int J Clin Exp Pathol. 2017;10(10):10419-10430.
48. Yao D-J, Qiao S, Zhang Y, Zhao Y-T, Yuan C-H. Correlation between expression of LRP16, Ki67 and EGFR and breast cancer clinical pathologic factors and prognosis. Eur Rev Med Pharm Sc. 2017; 21 (3S): 47-51.
49. [Giltnane JM,](https://www.ncbi.nlm.nih.gov/pubmed/?term=Giltnane%2520JM%255BAuthor%255D&cauthor=true&cauthor_uid=17634479) [Rydén L,](https://www.ncbi.nlm.nih.gov/pubmed/?term=Ryd%25C3%25A9n%2520L%255BAuthor%255D&cauthor=true&cauthor_uid=17634479) [Cregger M,](https://www.ncbi.nlm.nih.gov/pubmed/?term=Cregger%2520M%255BAuthor%255D&cauthor=true&cauthor_uid=17634479) [Bendahl PO,](https://www.ncbi.nlm.nih.gov/pubmed/?term=Bendahl%2520PO%255BAuthor%255D&cauthor=true&cauthor_uid=17634479) [Jirström K,](https://www.ncbi.nlm.nih.gov/pubmed/?term=Jirstr%25C3%25B6m%2520K%255BAuthor%255D&cauthor=true&cauthor_uid=17634479) [Rimm DL.](https://www.ncbi.nlm.nih.gov/pubmed/?term=Rimm%2520DL%255BAuthor%255D&cauthor=true&cauthor_uid=17634479) Quantitative measurement of epidermal growth factor receptor is a negative predictive factor for tamoxifen response in hormone receptor positive premenopausal breast cancer. [J Clin Oncol.](https://www.ncbi.nlm.nih.gov/pubmed/?term=Quantitative+measurement+of+epidermal+growth+factor+receptor+is+a+negative+predictive+factor+for+tamoxifen+response+in+hormone+receptor+positive+premenopausal+breast+cancer) 2007; 25(21): 3007-14.
50. [Qiao EQ,](https://www.ncbi.nlm.nih.gov/pubmed/?term=Qiao EQ%5BAuthor%5D&cauthor=true&cauthor_uid=24649232) [Ji M,](https://www.ncbi.nlm.nih.gov/pubmed/?term=Ji M%5BAuthor%5D&cauthor=true&cauthor_uid=24649232) [Wu J,](https://www.ncbi.nlm.nih.gov/pubmed/?term=Wu J%5BAuthor%5D&cauthor=true&cauthor_uid=24649232) [Li J,](https://www.ncbi.nlm.nih.gov/pubmed/?term=Li J%5BAuthor%5D&cauthor=true&cauthor_uid=24649232) [Xu X,](https://www.ncbi.nlm.nih.gov/pubmed/?term=Xu X%5BAuthor%5D&cauthor=true&cauthor_uid=24649232) [Ma R,](https://www.ncbi.nlm.nih.gov/pubmed/?term=Ma R%5BAuthor%5D&cauthor=true&cauthor_uid=24649232) [Zhang X,](https://www.ncbi.nlm.nih.gov/pubmed/?term=Zhang X%5BAuthor%5D&cauthor=true&cauthor_uid=24649232) [He Y,](https://www.ncbi.nlm.nih.gov/pubmed/?term=He Y%5BAuthor%5D&cauthor=true&cauthor_uid=24649232) [Zha Q,](https://www.ncbi.nlm.nih.gov/pubmed/?term=Zha Q%5BAuthor%5D&cauthor=true&cauthor_uid=24649232) [Song X,](https://www.ncbi.nlm.nih.gov/pubmed/?term=Song X%5BAuthor%5D&cauthor=true&cauthor_uid=24649232) [Zhu L,](https://www.ncbi.nlm.nih.gov/pubmed/?term=Zhu L%5BAuthor%5D&cauthor=true&cauthor_uid=24649232) [Tang JH.](https://www.ncbi.nlm.nih.gov/pubmed/?term=Tang JH%5BAuthor%5D&cauthor=true&cauthor_uid=24649232) Joint detection of multiple immunohistochemical indices and clinical significance in breast cancer. [Mol Clin Oncol.](https://www.ncbi.nlm.nih.gov/pubmed/24649232) 2013;1(4):703710.
51. Aziz SA, Pervez S, Khan S, Kayani N, Rahbar MH. Epidermal growth factor receptor (EGFR) as a prognostic marker: an immunohistochemical study on 315 consecutive breast carcinoma patients. J Pak Med Assoc. 2002 Mar;52(3):104-10. PMID: 12071064.
52. Aboushousha T, Hammam O, Safwat G, Eesa A, Ahmed S, Esmat ME, Helmy AH. Differential expression of RAGE, EGFR and Ki-67 in primary tumors and lymph node deposits of breast carcinoma. Asian Pac J Cancer Prev. 2018; 19 (8): 2269-2277.
53. Shashidhar HB, Lahari N A, Bharathi M. Diagnostic utility of VEGF in breast lesions. Indian J Pathol Oncol 2021;8(2):276-279.
54. Nakamura Y, Yasuoka H, Tsujimoto M, Yang Q, Tsukiyama A, Imabun S, Nakahara M, Nakao K, Nakamura M, Mori I, Kakudo K. Clinicopathological significance of vascular endothelial growth factor-C in breast carcinoma with long-term follow-up. Mod Pathol. 2003;16(4):309-14.
55. Almumen M. Immunohistochemical expression of VEGF in relation to other pathological parameters of breast carcinoma. J Cancer Ther. 2015; 6: 811-820.
56. Raghab H, Shaaban H, Maksoud N, Radwan S, Abd Elaziz W, Hafez N. Expression of vascular endothelial growth factor protein in both serum samples and excised tumor tissues of breast carcinoma patients. Int J Cancer Res. 2016; 12: 152-161.
57. Cˆımpean AM, Raica M, Suciu C, T˘atucu D, Sˆarb S, Mures¸an AM. Vascular endothelial growth factor A (VEGF A) as individual prognostic factor in invasive breast carcinoma. Rom J Morphol Embryol. 2008;49(3):303–308.
58. Jacobs EJ, Feigelson HS, Bain EB, Brady KA Rodriguez C. Polymorphisms in the vascular endothelial growth factor gene and breast cancer in the Cancer Prevention Study II cohort. Breast Cancer Res. 2006; 8(10):1186.
59. Schneider BP, Wang M, Radovich M, Sledge GW, Badve S, Thor A, Flockhart DA, Hancock B, Davidson N, Gralow J, et al. Association of vascular endothelial growth factor and vascular endothelial growth factor receptor-2 genetic polymorphisms with outcome in a trial of paclitaxel compared with paclitaxel plus bevacizumab in advanced breast cancer. J Clin Oncol. 2008; 26: 4672-4678.
60. Kidd LR, Brock GN, VanCleave TT, Benford ML, Lavender NA, Kruer TL, Wittliff JL. Angiogenesis-associated sequence variants relative to breast cancer recurrence and survival. Cancer Causes Control. 2010: 21: 1545–1557.
61. Su F, Liu B, Chen M, Xiao J, Li X, Lv X, Ma J, You K, Zhang J, Zhang Y. Association between VEGF-A, C and D expression and lymph node involvement in breast cancer: a meta-analysis. Int J Biol Markers. 2016;31(3):e235-44.
62. Sambyal V, Guleria K, Kapahi R, Manjari M, Sudan M, Uppal MS, Singh NR. Association of VEGF haplotypes with breast cancer risk in North-West Indians. BMC Med Genomics. 2021;14(1):209.
63. Ghasemi M, Emadian O, Naghshvar F, Bekhradnia A, Abediankenari S, Larijani LV, Moghimpour R. Immunohistochemical expression of vasculae endothelial growth factor and its correlation with tumor grade in breast ductal carcinoma. Acta Med Iran. 2011; 49(12): 776-779.
64. [Linardou H,](https://www.ncbi.nlm.nih.gov/pubmed/?term=Linardou%2520H%255BAuthor%255D&cauthor=true&cauthor_uid=23146280) [Kalogeras KT,](https://www.ncbi.nlm.nih.gov/pubmed/?term=Kalogeras%2520KT%255BAuthor%255D&cauthor=true&cauthor_uid=23146280) [Kronenwett R,](https://www.ncbi.nlm.nih.gov/pubmed/?term=Kronenwett%2520R%255BAuthor%255D&cauthor=true&cauthor_uid=23146280) [Kouvatseas G,](https://www.ncbi.nlm.nih.gov/pubmed/?term=Kouvatseas%2520G%255BAuthor%255D&cauthor=true&cauthor_uid=23146280) [Wirtz RM,](https://www.ncbi.nlm.nih.gov/pubmed/?term=Wirtz%2520RM%255BAuthor%255D&cauthor=true&cauthor_uid=23146280) [Zagouri F,](https://www.ncbi.nlm.nih.gov/pubmed/?term=Zagouri%2520F%255BAuthor%255D&cauthor=true&cauthor_uid=23146280) [Gogas H,](https://www.ncbi.nlm.nih.gov/pubmed/?term=Gogas%2520H%255BAuthor%255D&cauthor=true&cauthor_uid=23146280) [Christodoulou C,](https://www.ncbi.nlm.nih.gov/pubmed/?term=Christodoulou%2520C%255BAuthor%255D&cauthor=true&cauthor_uid=23146280) [Koutras AK,](https://www.ncbi.nlm.nih.gov/pubmed/?term=Koutras%2520AK%255BAuthor%255D&cauthor=true&cauthor_uid=23146280) [Samantas E,](https://www.ncbi.nlm.nih.gov/pubmed/?term=Samantas%2520E%255BAuthor%255D&cauthor=true&cauthor_uid=23146280) et al. The prognostic and predictive value of mRNA expression of vascular endothelial growth factor family members in breast cancer: a study in primary tumors of high-risk early breast cancer patients participating in a randomized Hellenic Cooperative Oncology Group trial. [Breast Cancer Res.](https://www.ncbi.nlm.nih.gov/pubmed/?term=The+Prognostic+and+Predictive+Value+of+mRNA+Expression+of+Vascular+Endothelial+Growth+Factor+Family+Members+in+Breast+Cancer%253A+A+Study+in+Primary+Tumors+of+High-Risk+Early+Breast+Cancer+Patients+Participating+in+a+Randomized+Hellenic+Cooperative+Oncology+Group+Trial) 2012; 14(6): R145.
65. Srabovic N, Mujagic Z, Mujanovic-Mustedanagic J, Softic A, Muminovic Z, Rifatbegovic A, Begic L. Vascular endothelial growth factor receptor-1 expression in breast cancer and its correlation to vascular endothelial growth factor A. Int J Breast Cancer 2013:746749.
66. Fuckar D, Dekanic A, Stifter S, Mustac E, Krstulja M, Dobrila F, Jonjic N. VEGF expression is associated with negative estrogen receptor status in patients with breast cancer. Int J Surg Pathol. 2006; 14:49–55.
67. Liu Y, Tamimi RM, Collins LC, Schnitt SJ, Gilmore HL, Connolly JL, Colditz GA. The association between vascular endothelial growth factor expression in invasive breast cancer and survival varies with intrinsic subtypes and use of adjuvant systemic therapy: results from the Nurses’ Health Study. Breast Cancer Res Treat. 2011; 129(1):175–184.
68. Linderholm B, Bergqvist J, Hellborg H, Johansson U, Linderholm M, von Schoultz E, Elmberger G, Skoog L, Bergh J. Shorter survival-times following adjuvant endocrine therapy in oestrogenand progesterone-receptor positive breast cancer overexpressing HER2 and/or with an increased expression of vascular endothelial growth factor. Med Oncol. 2009; 26:480–490.
69. [Zhu Y,](https://www.ncbi.nlm.nih.gov/pubmed/?term=Zhu%2520Y%255BAuthor%255D&cauthor=true&cauthor_uid=26531769) [Guo M,](https://www.ncbi.nlm.nih.gov/pubmed/?term=Guo%2520M%255BAuthor%255D&cauthor=true&cauthor_uid=26531769) [Zhang L,](https://www.ncbi.nlm.nih.gov/pubmed/?term=Zhang%2520L%255BAuthor%255D&cauthor=true&cauthor_uid=26531769) [Xu T,](https://www.ncbi.nlm.nih.gov/pubmed/?term=Xu%2520T%255BAuthor%255D&cauthor=true&cauthor_uid=26531769) [Wang L,](https://www.ncbi.nlm.nih.gov/pubmed/?term=Wang%2520L%255BAuthor%255D&cauthor=true&cauthor_uid=26531769) [Xu G.](https://www.ncbi.nlm.nih.gov/pubmed/?term=Xu%2520G%255BAuthor%255D&cauthor=true&cauthor_uid=26531769) Biomarker triplet NAMPT/VEGF/HER2 as a de novo detection panel for the diagnosis and prognosis of human breast cancer. [Oncol Rep.](https://www.ncbi.nlm.nih.gov/pubmed/?term=Biomarker+triplet+NAMPT%252FVEGF%252FHER2+as+a+de+novo+detection+panel+for+the+diagnosis+and+prognosis+of+human+breast+cancer) 2016; 35(1): 454-62.
70. Chen Z, Xu S, Xu W, Huang J, Zhang G, Lei L, Shao X, Wang X. Expression of cluster of differentiation 34 and vascular endothelial growth factor in breast cancer, and their prognostic significance. Oncology Letters. 2015; 10: 723-729.
71. Mohammed RA, Green A, El-Shikh S, Paish EC, Ellis IO, Martin SG. Prognostic significance of vascular endothelial cell growth factors -A, -C and -D in breast cancer and their relationship with angio- and lymphangiogenesis. Br J Cancer. 2007; 96(7):1092-100.
72. Comsa S, Compean AM, Ceausu R, Suciu C, Raica R. Correlations between vascular endothelial growth facor expression, microvascular density in tumor tissues and TNM staging in breast cancer. Arch Biol Sci. 2012; 64: 409-417.
73. Halimi M, Vahedi A, Mostafapour EK. Association of VEGF with Regional Lymph Node Metastasis in Breast IDC. J Med Sci. 2012; 12:18-23.
74. Shankar R, Tiwary SK, Khanna R, Kumar M, Khanna AK. Tumor angiogenesis: determined by vegf expression, mags scoring, doppler study, as prognostic indicator in carcinoma breast. Internet J Surg. 2006; 8(1): 5.
75. Al-Bassam SSA, Kadhim HS, Khashman BM. Possible association of vascular endothelial growth factor with grades of breast cancer. Int J Res Pharm Chem. 2014; 4: 291-293.
76. Ellis L. Epidermal growth factor receptor in tumor angiogenesis. Hematol Oncol Clin North Am. 2004; 18: 1007 – 21.
77. Hanahan D, Weinberg RA. Hallmarks of cancer: the next generation. Cell. 2011; 144(5): 646-74
78. Lee T-H, Seng S, Sekine M, Hinton C, Fu Y, Avraham HK, Avraham S. Vascular endothelial growth factor mediates intracrine survival in human breast carcinoma cells through internally expressed VEGFR1/FLT1. PLoS Medicine. 2007; 4(6): e186.
79. Miettinen M., Rikala M-S., Rysz J., Lasota J. & Wang Z-F. Vascular endothelial growth factor receptor 2 (VEGFR2) as a marker for malignant vascular tumors and mesothelioma-immunohistochemical study of 262 vascular endothelial and 1640 nonvascular tumors. Am J Surg Pathol 2012; 36 (4): 629-639.
80. Nasir A, Holzer TR, Chen M, Man MZ, Schade AE. Differential expression of VEGFR2 protein in HER2 positive primary human breast cancer: potential relevance to anti‑angiogenic therapies. Cancer Cell Int. 2017; 17:56.
