# Supplementary material for: Epidermal growth factor receptor (EGFR) and vascular endothelial growth factor A (VEGF-A) expressions in Ethiopian female breast cancer and their association with histopathologic features
Source: PLoS One. 2024 Oct 15;19(10):e0308411. doi: 10.1371/journal.pone.0308411 (PMC11478813; doi:10.1371/journal.pone.0308411)
Supplement: Attachment — Submitted filename: Response to reviewers.doc [file pone.0308411.s002.doc]

**Response to Reviewers**

Editor-in-Chief

PLOS ONE

We would like to express our sincere gratitude to the reviewers for their valuable and constructive assessment of our paper. Below you will find our specific responses to the reviewer’s comments.

**General comments of reviewer 4**

Comment 1: The method section should be clear, specially, IHC scoring, inclusion and exclusion criteria?

Response 1: Within the manuscript, we tried to correct the methodology and clarified it based on the comments given.

Comment 2: Some percentage disagreement in the result should be revised.

Response 2: The disagreement arose due to errors in writing the data, which have now been included and corrected.

Comment 3: Better to indicate the scientific and clinical value of these markers rather than simple comparison with other results. All necessary comments are uploaded in the word document.

Response 3: As much as we can, apart from comparison, we added some clinical values of these markers. We corrected based on the comments given in the word document.

**Specific comments of reviewer 4 on the word document (manuscript)**

1. **Title**

Comment 1: **Don’t use abbreviation in the title, if possible, try to indicate your main finding in the title.**

Response: Abbreviations are removed from the title.

Regarding title, we can change the title to “Epidermal growth factor receptor (EGFR) but not vascular endothelial growth factor A is associated with ER and PR status in Ethiopian female breast cancer cases”. However, we felt that the previous title include all the variables studied.

2. **Abstract section**

Comment 2: Why you only select modified radical mastectomy samples?

Response: Modified radical mastectomy is the type of surgery done in the hospitals for breast cancer here in the Ethiopian Hospitals.

Comment 3: Better to avoid abbreviation at the beginning of sentence in the whole text.

Abbreviations at the beginning of the paragraphs are avoided from the whole document.

3. **Introduction part**

Comment 4: Try to include targeted treatment for triple negative breast cancer patients.

Response: Some targeted treatments for triple negative patients are included with citations in the first paragraph of the introduction part.

Comment 5: It is difficult to say this, you have to make sure about this sentence! Maybe it is better to link with VEGF and EGFR

Response: The sentence is modified.

4. **Materials and methods part**

Comment 6: Specific ethical number for both review board is needed.

Response: In the ethical considerations, ethical numbers are included for the ethical approval letters of both review boards.

Comment 7: Names of hospitals? Where did resection was done?

Response: Name of the hospitals and the places of the tissue resection are specified.

Comment 8: What are the other inclusion and exclusion criteria?

Response: Additional inclusion and exclusion criteria are included.

Comment 9: High or low PH buffer for antigen retrieval?

Response: pH for antigen retrieval is indicated.

Comment 10: Did all slides scan or only EGFR and VEGF? Is the scoring microscopically or software based?

Response: Scanning procedure for which slides is clarified.

Comment 11: Have you scored nuclear, cytoplasmic or membranous staining or all?

Response: Scoring criteria is specified.

5. **Results part**

Comment 12: Better to include mean age and age range in table 1

Response: Mean age and age range are added in the table.

Comment 13: Why? Since you have used MRM samples?

Response: The reason is specified.

Comment 14: It is not 85(83+2 in the table), 17+6+1+6+48=78, where is the remaining 5 samples?

Response: The missing numbers are included and corrected.

Comment 15: Though it is not significant, better to indicate in the text result.

Response: It is indicated in the text based on the comment.

Comment 16: Indicate the p-value of age vs VEGF status in text.

Response: It is indicated in the text based on the comment.

6. **Discussion part**

Comment 17: Better to have the summary main result here?

Response: It is added in the paragraph based on the comment.

Comment 18: Look additional reason for variation in EGFR expression between current finding and previous studies.

Response: Additional reasons are included based on the comments.

Comment 19: It is higher in older age group in your result, though it not significant? Discuss it!

Response: The reasons for higher VEGF expressions at older age are discussed.

Comment 20: Correct the limitation part

Response: Limitation statement is modified.

7. **Conclusion part**

Comment 21: Clarify the statement in the conclusion.

Response: The statement is clarified

8. **Declaration part**

Comment 22: Please delete the one of the ethical consderations (either from method or here), but include the approval number?

Response: Ethical considerations is deleted from here based on the comment given by you and by the editor. It is included in the methods part. Ethical letter numbers of both institutions are indicated.

Sincerely

Sisay Addisu (PhD)

Corresponding author
